# High-Accuracy Indoor Multiple-Extended-Target Tracking Algorithm Based on 60 GHz Millimeter-Wave Radar

**DOI:** 10.3390/s26123758

**Published:** 2026-06-12

**Authors:** Bo Gao, Jianzhong Chen, Bo Huang, Geng Yang

**Affiliations:** 1 College of Information and Control Engineering, Xi’an University of Architecture and Technology, Xi’an 710055, China; 2 Faculty of Electronic and Information Engineering, Xi’an Jiaotong University, Xi’an 710049, China

**Keywords:** millimeter-wave radar, multiple-extended-target tracking, Density-Based Spatial Clustering of Applications with Noise, Extended Kalman Filter, Nearest-Neighbor Data Association

## Abstract

The rapid development of Internet of Things technologies has accelerated the deployment of smart home systems. However, perception solutions based on visual sensors remain constrained by illumination sensitivity, occlusion, and privacy concerns. Frequency-modulated continuous-wave (FMCW) millimeter-wave radar provides a promising alternative because it operates independently of lighting conditions, is robust to environmental changes, and preserves user privacy. To address multiple-extended-target tracking in cluttered indoor environments, this paper proposes a high-accuracy tracking algorithm that combines an improved Density-Based Spatial Clustering of Applications with Noise (DBSCAN) algorithm, an optimized Nearest-Neighbor Data Association (NNDA) scheme, and an Extended Kalman Filter (EKF). The improved DBSCAN algorithm introduces spatial-extent constraints, velocity-consistency checks, and candidate-cluster validation to cluster raw radar point clouds and convert extended targets into representative point targets with little additional computational cost. The optimized NNDA scheme then integrates clustering information into the association process, improving the matching accuracy between existing tracks and current measurements. Finally, the EKF estimates the state of each target from the associated measurements. Real-world experiments show that the proposed algorithm achieves tracking errors below 0.4 m in typical motion scenarios, maintains continuous tracking in two-person crossing scenarios, and reaches 93.3% counting accuracy in five-person scenarios. These results outperform the tracking system based on the commercial Texas Instruments (TI) IWR6843ISK millimeter-wave radar evaluation board. The proposed method offers a reliable and privacy-preserving sensing solution for smart homes, elderly care, and intelligent building applications.

## 1. Introduction

In recent years, the rapid development of indoor intelligent technologies has made indoor target tracking an important research topic in intelligent perception. In indoor environments, accurate tracking of people, objects, and animals provides essential technical support for target management and intelligent services in smart homes [1], smart healthcare [2], industrial automation [3], and related applications.

Conventional indoor perception systems are commonly based on optical cameras, infrared sensors, ultrasonic sensors [4], or wearable devices. Nevertheless, these technologies have clear limitations in complex indoor environments. Depth cameras are prone to recognition errors in the event of severe occlusion [5]; RGB cameras are sensitive to illumination changes and cluttered backgrounds [6] while also raising privacy concerns [7]; thermal infrared cameras [8] usually provide only coarse human detection and limited target details; and wearable devices such as smart bracelets [9] can capture only local motion information and cannot support full-scene tracking.

Millimeter-wave radar technology [10] has emerged as a promising alternative means of indoor perception. First, its high operating frequency enables high-resolution perception and the capture of subtle target motions. Second, millimeter waves can partially penetrate common non-metallic materials, such as wood, drywall, and fabrics, allowing reliable detection even when targets are partially occluded by household objects. Third, radar sensing is independent of lighting conditions and remains effective in dark or low-light environments. Fourth, millimeter-wave radar does not acquire images, thereby reducing the risk of privacy leakage associated with vision-based systems. These advantages make indoor multi-target tracking with millimeter-wave radar both theoretically valuable and practically important for intelligent indoor sensing.

## 2. Related Work

### 2.1. Millimeter-Wave Radar Technology

Research on millimeter-wave radar technology outside China can be traced back to the 1950s, when studies of millimeter-wave propagation supported early applications such as airport traffic control [11]. In the 1970s, the technology was mainly driven by military requirements and was applied to missile guidance and battlefield reconnaissance [12]. By the 1990s, advances in semiconductor technology promoted the transition of millimeter-wave radar systems from discrete components to integrated architectures. Gallium arsenide (GaAs) technology became widely used, and improvements in transmitter, receiver, and antenna design laid the foundation for later civilian applications [13,14].

Since the beginning of the 21st century, millimeter-wave radar has been increasingly adopted in automotive and industrial applications. In 2007, silicon–germanium (SiGe) technology gradually replaced GaAs in many radio-frequency front ends, improving chip integration and reducing system cost. With the rapid development of autonomous driving, millimeter-wave radar has become one of the key sensors in advanced driver-assistance systems [15]. International automotive electronics manufacturers such as Bosch, Continental, and Delphi have released high-performance radar products for adaptive cruise control, forward collision warning, blind-spot detection, and related functions [16,17].

In China, millimeter-wave radar research started later, but it has developed rapidly in recent years with national support for intelligent connected vehicles and smart sensing technologies. Universities, research institutes, and enterprises have made notable progress in core device development, system architecture, and industrial implementation.

Representative studies illustrate this progress. In 2020, Shi investigated antenna array design for millimeter-wave MIMO radar and broadband vehicular radar arrays, improving resolution and detection performance [18]. In 2021, Ji studied vital sign detection based on millimeter-wave radar and demonstrated non-contact monitoring of human cardiopulmonary activity, providing a potential sensing method for healthcare applications [19]. In 2022, Wang examined millimeter-wave radar for advanced driver-assistance systems from the perspective of business model innovation and discussed differentiated product positioning, cooperative networks, and profit models for commercialization [20]. In 2023, Gong proposed an OTFS-modulation-based radar parameter estimation algorithm that improved range, velocity, and angle estimation accuracy, and they further developed super-resolution and interference-suppression methods for automotive radar scenarios [21]. In 2025, a joint team from Nankai University and the City University of Hong Kong reported an integrated thin-film lithium niobate photonic millimeter-wave radar chip using a CMOS-compatible process, achieving centimeter-level inverse synthetic aperture imaging and showing potential for 6G communication and intelligent driving.

### 2.2. Multiple-Extended-Target Tracking Algorithms

Multi-target tracking generally consists of point cloud clustering, motion modeling, data association, and filtering-based state estimation. Among these steps, clustering and data association are particularly important for extended targets because each physical target may generate multiple radar points in each frame. This section therefore reviews the application of millimeter-wave radar to multiple-extended-target tracking from the perspectives of clustering and data association.

#### 2.2.1. Clustering Algorithms

Clustering algorithms directly affect both tracking accuracy and computational efficiency. Since MacQueen introduced the K-means algorithm in the 1960s, clustering has become a major research topic [22]. In the late 1970s, Dempster et al. proposed the expectation-maximization (EM) algorithm, which provides an effective approach for maximum likelihood estimation with latent variables [23]. In the early 1990s, Kaufman and Rousseeuw proposed the partitioning around medoids (PAM) algorithm to reduce the sensitivity of K-means to outliers by using dissimilarity measures and representative medoids [24]. In the late 1990s, Ester et al. proposed DBSCAN, a density-based algorithm that can identify clusters of arbitrary shape and is robust to noise [25].

Recent studies have further adapted DBSCAN and related clustering methods to radar point clouds. In 2015, Wagner et al. introduced size parameters into DBSCAN and developed an ellipsoidal DBSCAN method for pedestrian identification using range, Doppler, and angle-of-arrival measurements in automotive radar systems [26]. In 2016, Zhou et al. proposed AF-DBSCAN, which combines K-nearest-neighbor distributions with statistical analysis to estimate the global parameters Eps and MinPts automatically [27]. In 2021, Ju et al. proposed a radar-adaptive DBSCAN algorithm that adjusts the clustering radius according to K-nearest-neighbor distance and target radar cross-section, improving pedestrian and vehicle recognition accuracy [28]. In 2023, Peng et al. addressed the sparsity and nonuniformity of indoor millimeter-wave radar point clouds by proposing a joint density- and partition-based clustering method [29]. In 2024, Chen et al. proposed an adaptive DBSCAN algorithm combining Mahalanobis and Euclidean distances to reduce over-segmentation and false alarms in road-scene radar point clouds [30].

#### 2.2.2. Data Association Algorithms

Data association is another core component of multi-target tracking [31]. Its primary task is to establish correspondences between current sensor measurements and existing target tracks, thereby determining which target each measurement most likely belongs to. Commonly used data association algorithms include Nearest-Neighbor Data Association (NNDA), probabilistic data association (PDA), and joint probabilistic data association (JPDA).

In 1971, Singer et al. proposed the NNDA algorithm, which associates a target with the measurement closest to its predicted position within a validation gate. Because of its low computational cost and simple implementation, NNDA has been widely used in automotive sensing systems [32]. In 1975, Bar-Shalom and Tse proposed PDA, which calculates the posterior association probability for each observation within the validation gate. However, PDA assumes that each track is isolated, and so its reliability decreases in dense multi-target environments [33]. In 1974, Bar-Shalom proposed JPDA as a Bayesian method for multi-target tracking. Although JPDA achieves better association robustness than PDA, the number of joint association hypotheses grows rapidly with the number of targets and measurements, causing combinatorial explosion and unacceptable latency in many engineering applications [34].

Recent studies have attempted to improve association accuracy and real-time performance. In 2018, Zhang et al. used a nearest-neighbor strategy with parallel interacting multiple-model filters to improve the real-time performance and stability of radar flight-inspection tracking [35]. In 2020, Han et al. proposed an NNDA method based on a dynamic target-monitoring model. After a gate search and target updating, NNDA is used for association and track updating; although it is slower than standard NNDA, it is significantly faster than multi-hypothesis tracking (MHT) [36]. In 2021, Zheng et al. proposed a variational-inference-based JPDA algorithm for radar multi-target tracking, reducing mis-tracking in dense scenarios by modeling measurement errors probabilistically [37]. In 2022, Li et al. proposed an adaptive-threshold NNDA method in which the association threshold is calculated from the correlation values of measurements within the validation gate [38].

Despite these advances, existing clustering and association methods still have limitations in indoor scenes with high target density, close spacing, occlusion, clutter, and multiple paths. In such cases, traditional methods are prone to target loss, false association, and trajectory drift. Improving the separation of multiple extended targets and maintaining stable real-time tracks therefore remain key challenges for indoor millimeter-wave radar perception.

### 2.3. Main Work

Motivated by these challenges, this paper proposes a complete millimeter-wave radar system for indoor tracking of multiple extended targets. This study’s main contributions to the literature are as follows. First, an improved DBSCAN clustering algorithm is developed by introducing spatial-extent constraints, velocity-consistency checks, and SNR-based candidate validation. This design reduces the merging of point clouds from closely spaced human targets and suppresses false clusters caused by clutter or multipath effects. Second, an optimized NNDA scheme is proposed to integrate clustering and association more tightly, thereby improving association robustness while maintaining low computational complexity. Third, the proposed pipeline is implemented and validated on a 60 GHz FMCW radar platform consisting of a TI IWR6843ISK evaluation module and a DCA1000EVM data-capture board, demonstrating its effectiveness in complex indoor scenarios.

The remainder of this paper is organized as follows. Section 3 introduces the fundamental principles of radar target tracking, including radar data preprocessing, track initiation, track filtering, and track management. Section 4 presents the proposed indoor multi-extended-target tracking algorithm, including the improved DBSCAN clustering algorithm, the optimized NNDA association scheme, and the EKF-based tracking procedure. Section 5 evaluates the proposed system through real-world experiments and compares it with existing methods. Section 6 concludes the paper.

## 3. Fundamental Principles of Radar Target Tracking

This section details the fundamental principles and components that form the basis of the radar target-tracking pipeline used in this work. It covers data preprocessing, track initiation, and the core track-filtering process.

### 3.1. Radar Data Preprocessing

The processed output of the FMCW millimeter-wave radar is a set of detection points. Each point is first represented in the radar local polar coordinate system, including range, azimuth, and elevation, because this is the native measurement domain of range–Doppler–angle processing [39]. These measurements are then transformed into a Cartesian coordinate frame for subsequent filtering, association, and tracking. The data preprocessing module removes invalid or interfering points, performs coordinate transformation, and standardizes the data format. It provides reliable input for the subsequent modules and consists of two main stages: coordinate transformation and point-cloud filtering.

#### 3.1.1. Coordinate Transformation

**Figure 1:**
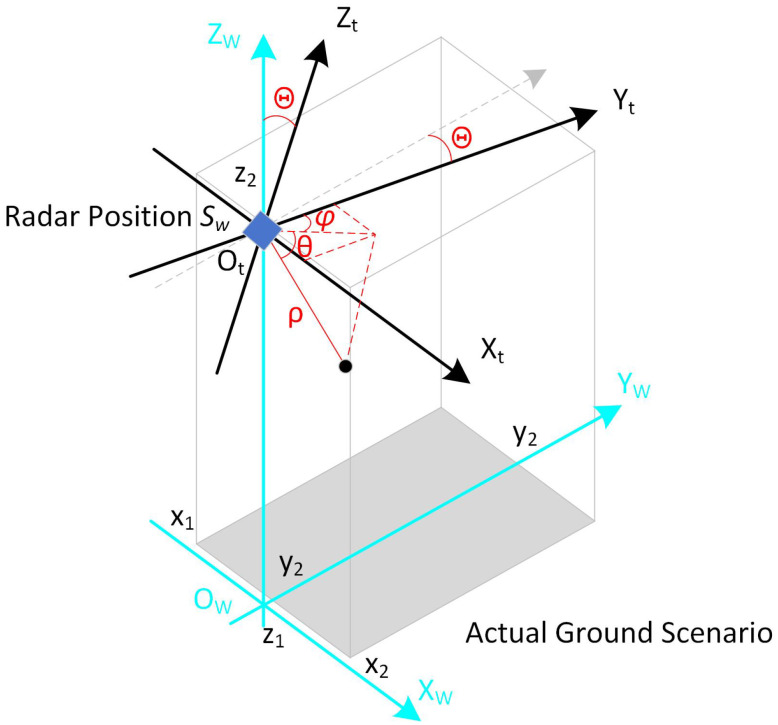
Schematic diagram of radar 3D coordinate transformation.

Because radar measurements are obtained in polar coordinates, the original point cloud is expressed in a local polar coordinate system whose origin is the radar antenna phase center. For tracking in a room-level coordinate frame, the measurements are first converted from local polar coordinates to local Cartesian coordinates and then transformed into the global Cartesian coordinate system. Figure 1 illustrates the relationship among the local polar coordinate system, the radar-aligned local Cartesian coordinate system, and the room-fixed global Cartesian coordinate system [39]. These transformations, summarized in Table 1, enable all measurements and tracks to be represented in a common reference frame.

For 3D radar detection, each measurement is expressed as [ρ,ϕ,θ], as shown in Figure 1. Here, ρ denotes the radial distance from the detection point to the radar antenna, ϕ denotes the azimuth angle in the horizontal projection plane, and θ denotes the elevation angle relative to the horizontal plane of the radar system.

If the radar rotates clockwise around the $X_{w}$-axis by an angle of Θ, the rotation matrix is

(1) $$R_{x}\left(\Theta \right)=\left[\begin{array}{ccc} 1 & 0 & 0\\ 0 & \cos\Theta & \sin\Theta \\ 0 & -\sin\Theta & \cos\Theta \end{array}\right]$$

Suppose that point *P* has local polar coordinates [ρ,ϕ,θ] and local Cartesian coordinates $\left[x_{t},y_{t},z_{t}\right]$. The transformation from the local polar coordinate system to the local Cartesian coordinate system is

(2) $$\left\{\begin{array}{c} x_{t}=\rho \cos\theta \sin\phi \\ y_{t}=\rho \cos\theta \cos\phi \\ z_{t}=\rho \sin\theta \end{array}\right.$$

Finally, the transformation from the local Cartesian coordinate system to the global coordinate system is

(3) $$\left[\begin{array}{c} x_{w}\\ y_{w}\\ z_{w} \end{array}\right]=R_{x}\left(\Theta \right)\left[\begin{array}{c} x_{t}\\ y_{t}\\ z_{t} \end{array}\right]+\left[\begin{array}{c} 0\\ 0\\ H_{r} \end{array}\right]$$

#### 3.1.2. Point-Cloud Filtering

Point-cloud filtering screens the transformed point cloud to remove invalid points and reduce noise before tracking. The process consists of two sequential stages:

1. Spatial filtering: A valid tracking region is predefined as a 2D boundary in the global Cartesian coordinate system. Points whose transformed global coordinates (x,y) lie inside this boundary are retained, whereas points outside the region of interest are treated as interference and discarded.
2. Static point filtering: After spatial filtering, a Doppler velocity check is applied. Points whose measured radial velocity magnitude |v| is below a predefined threshold are regarded as static or near-static points. These points usually correspond to stationary clutter or very slow objects and are separated from the moving-target tracking pipeline.

### 3.2. Track Initiation

Track initiation is a prerequisite for multi-target tracking. It establishes a stable track from scattered measurements after a new target enters the radar detection region [39]. The main objectives are to identify real targets quickly while suppressing false tracks. This process mainly involves tracking gate design and the track initiation algorithm.

#### 3.2.1. Tracking Gate

A tracking gate is a validation region used to determine whether a radar measurement may belong to a target. It is centered on the initial or predicted target position and bounded by the expected measurement uncertainty. The gate should be large enough to include true target measurements with high probability but small enough to reduce the probability of accepting measurements from other targets or clutter. Its size is affected by model prediction error, radar measurement error, target maneuverability, and physical target size.

**Figure 2:**
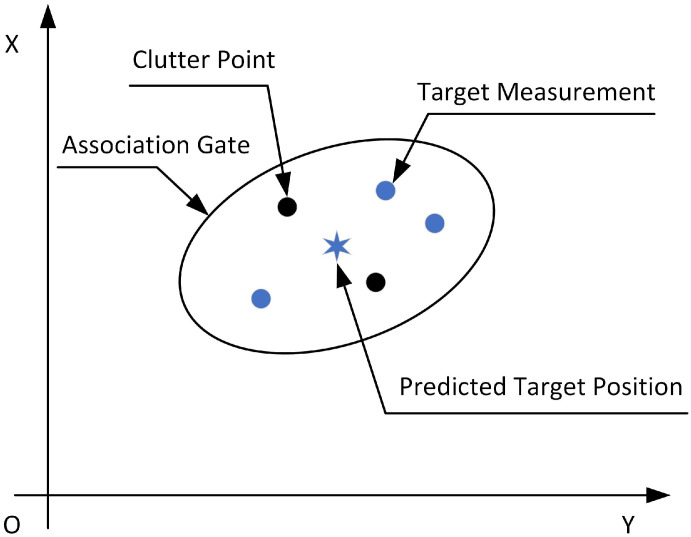
Schematic diagram of an elliptical gate.

According to the characteristics of indoor extended targets, this paper adopts an elliptical gate. The gate is centered on the predicted target position, as shown in Figure 2. Points inside the gate are candidate measurements; they may originate from the target itself, nearby targets, or interference.

If the measured value Z(k+1) in the Cartesian coordinate system satisfies

(4) $$D^{2}≜{\left(Z\left(k+1\right)-\hat{Z}\left(k+1|k\right)\right)}^{⊤}S^{-1}\left(k+1\right)\left(Z\left(k+1\right)-\hat{Z}\left(k+1|k\right)\right)\leq G$$

then the measured value Z(k+1) falls within the elliptical gate. Here, $D^{2}$ is the normalized innovation squared, a statistical measure of the distance between the measurement and the prediction. A measurement is considered to fall within the elliptical tracking gate if $D^{2}$ is less than or equal to a threshold *G*, which is derived from the chi-square distribution. $\hat{Z}\left(k+1|k\right)$ represents the predicted value of the target at the current time, Z(k+1) represents the measured value at the current time, $Z\left(k+1\right)-\hat{Z}\left(k+1|k\right)$ represents the innovation, and S(k+1) represents the innovation covariance matrix.

If the radar measurement vector is $z={\left[\rho ,\phi ,v\right]}^{⊤}$ and $\sigma_{\rho }^{2}$, $\sigma_{\phi }^{2}$ and $\sigma_{v}^{2}$ are the variances of the target’s range, azimuth angle, and velocity measurement errors, respectively, then the innovation covariance matrix is

(5) $$S=\left[\begin{array}{ccc} \sigma_{\rho }^{2} & 0 & 0\\ 0 & \sigma_{\phi }^{2} & 0\\ 0 & 0 & \sigma_{v}^{2} \end{array}\right]$$

Substituting into Equation (4), we obtain

(6) $$D^{2}=\frac{{\left(\rho -\hat{\rho }\right)}^{2}}{\sigma_{\rho }^{2}}+\frac{{\left(\phi -\hat{\phi }\right)}^{2}}{\sigma_{\phi }^{2}}+\frac{{\left(v-\hat{v}\right)}^{2}}{\sigma_{v}^{2}}$$

Since $\frac{\left(\rho -\hat{\rho }\right)}{\sigma_{\rho }}∼N\left(0,1\right)$, $\frac{\left(\phi -\hat{\phi }\right)}{\sigma_{\phi }}∼N\left(0,1\right)$, $\frac{\left(v-\hat{v}\right)}{\sigma_{v}}∼N\left(0,1\right)$ and they are independent of each other, the random variable $D^{2}$ follows a chi-square distribution with degrees of freedom n=3, i.e., $y=D^{2}∼\chi_{n}^{2}$. Its probability density function is

(7) $$f\left(y\right)=\frac{y^{\frac{n}{2}-1}}{2^{\frac{n}{2}}\Gamma \left(\frac{n}{2}\right)}e^{-\frac{y}{2}},\;y>0$$

where Γ(·) is the gamma function.

#### 3.2.2. Track Initiation Algorithm

Track initiation converts unassociated measurements from consecutive radar frames into initial target-track hypotheses. The algorithm links measurements across time according to motion consistency and spatial proximity, thereby determining whether a persistent target has appeared.

Indoor targets usually move slowly and exhibit smooth trajectory changes, making lightweight logic-based initiation suitable for real-time processing. This paper adopts a sliding-window m/n logic method because it has low computational cost, fast response, and good false-track suppression.

In this method, a time window containing *n* radar scans is maintained. If a measurement is detected within the relevant gate in a scan, the association result for that scan is recorded as 1; otherwise, it is recorded as 0. When at least *m* out of the *n* records are equal to 1, track initiation is declared successful. By selecting appropriate values of *m* and *n*, the system balances initiation speed and stability in indoor multi-target scenarios.

### 3.3. Track Filtering

Track filtering is the core step in target tracking. It estimates the true position, velocity, and acceleration of a target from noisy measurements, thereby suppressing measurement noise and smoothing the resulting trajectory. This part mainly includes target motion modeling and filtering algorithm selection.

#### 3.3.1. Target Motion Model

Indoor human motion is generally random and low-speed with a moderate degree of maneuvering. A conventional constant-velocity (CV) model cannot fully describe changes caused by walking, turning, and stopping. A constant-acceleration (CA) model is therefore adopted to represent indoor human motion more accurately. For the 2D tracking scenario considered here, the target state vector is defined as

(8) $$X={\left[x,\dot{x},\ddot{x},y,\dot{y},\ddot{y}\right]}^{T}$$

where *x* and *y* are position coordinates, $\dot{x}$ and $\dot{y}$ are velocity components, and $\ddot{x}$ and $\ddot{y}$ are acceleration components. Let the sampling interval be T; then, the state transition matrix *F* for the CA model is

(9) $$F=\left[\begin{array}{cccccc} 1 & T & T^{2}/2 & 0 & 0 & 0\\ 0 & 1 & T & 0 & 0 & 0\\ 0 & 0 & 1 & 0 & 0 & 0\\ 0 & 0 & 0 & 1 & T & T^{2}/2\\ 0 & 0 & 0 & 0 & 1 & T\\ 0 & 0 & 0 & 0 & 0 & 1 \end{array}\right]$$

where *F* propagates the state vector (containing position, velocity, and acceleration in *x* and *y*) from one time step to the next, assuming acceleration remains constant over the sampling interval *T*.

#### 3.3.2. Extended Kalman Filter

Millimeter-wave radar measurements are expressed in polar coordinates, whereas the target state is represented in Cartesian coordinates. The mapping between the state and the measurement is nonlinear, and so a standard Kalman filter cannot be applied directly. This paper therefore uses the EKF, which linearizes the nonlinear measurement function through a first-order Taylor expansion and provides accurate state estimation with moderate computational cost and good suitability for embedded implementation [40]. The EKF consists of two main steps: state prediction and measurement updating. Their closed-loop recursion produces a smoothed estimate of the target state.

State Prediction: The current target state and covariance are predicted from the optimal estimate at the previous time step,

(10) $$\begin{array}{cc} \hat{X}\left(k|k-1\right) & =F\hat{X}\left(k-1|k-1\right)\\ P(k|k-1) & =FP\left(k-1|k-1\right)F^{T}+Q \end{array}$$

where *P* is the state covariance matrix and *Q* is the process noise covariance matrix.

Measurement Updating: The predicted state is corrected using the current measurement. First, the nonlinear measurement function is linearized to obtain the Jacobian matrix *H*. The innovation, innovation covariance matrix, and Kalman gain are then calculated as follows:

(11) $$\begin{array}{cc} v(k) & =z\left(k\right)-\hat{z}\left(k|k-1\right)\\ S(k) & =HP\left(k|k-1\right)H^{T}+R\\ K(k) & =P\left(k|k-1\right)H^{T}S{\left(k\right)}^{-1} \end{array}$$

where *R* is the measurement noise covariance matrix. Finally, the state and covariance are updated as follows:

(12) $$\begin{array}{cc} \hat{X}\left(k|k\right) & =\hat{X}\left(k|k-1\right)+K\left(k\right)v\left(k\right)\\ P(k|k) & =(I-K(k)H)P(k|k-1) \end{array}$$

where *I* is the identity matrix.

#### 3.3.3. Track Management

Track management controls the life cycle of each target track. The main track states include the tentative, steady, and deleted track states.

**Figure 3:**
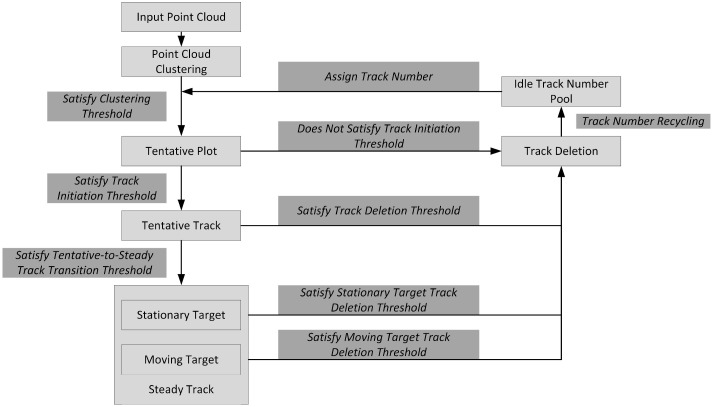
Track state transition diagram.

For extended-target tracking, the input radar point cloud is first clustered. A newly detected cluster is initially treated as a tentative plot. If it can be associated and updated consistently over consecutive frames and satisfies the initiation threshold, it is promoted to a tentative track. When the environment contains many clutter points, the initiation threshold can be increased to reduce false tracks. When clutter is limited, the threshold can be decreased to improve initiation speed. After track-to-measurement association, both tentative and steady tracks are updated. A tentative track is promoted to a steady track if it is successfully updated for at least *m* out of *n* consecutive scans. This m/n logic confirms persistent targets while suppressing transient false tracks caused by noise or clutter. If a track is successfully associated with point clouds, its current state is maintained. If it fails to be associated for several consecutive frames, it is deleted and its track number is recycled. In indoor tracking, a missing association may also occur when a person becomes stationary and the radar no longer detects moving points from that target, even though the target remains in the scene. For this reason, stationary-target tracks should use a larger deletion threshold to avoid premature deletion. The state transition process is shown in Figure 3.

## 4. Indoor Multiple-Extended-Target Tracking Algorithm

This section presents the core algorithmic contributions of this work. It details the overall tracking pipeline, followed by the proposed improved DBSCAN clustering and optimized NNDA association algorithms. Their integration and the final track prediction/updating steps are also described.

### 4.1. Overall Flow of the Tracking Algorithm

**Figure 4:**
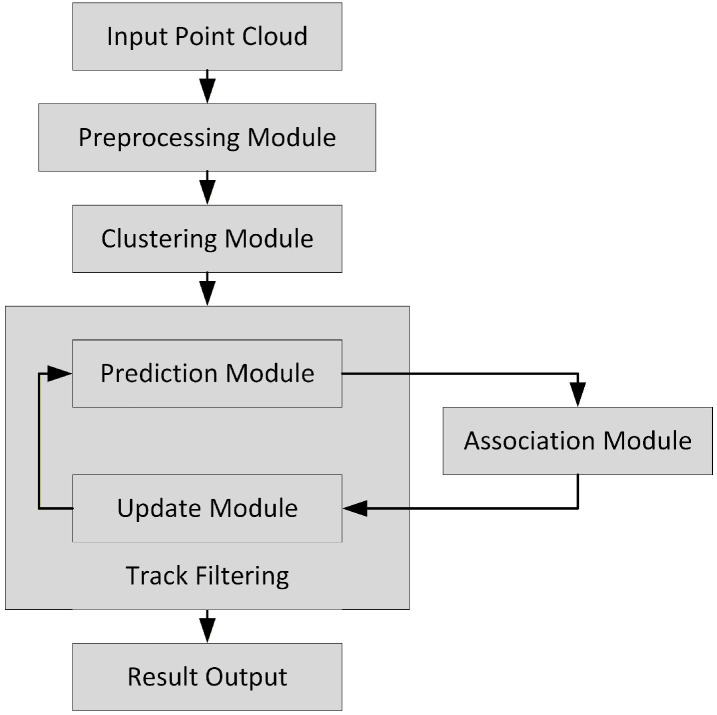
Overall flow of the tracking algorithm.

To address clutter interference, target occlusion, and data association errors in indoor multiple-extended-target tracking, the overall algorithm flow is designed as shown in Figure 4. The raw point-cloud output from the millimeter-wave radar is first preprocessed. The clustering module then converts extended-target point clouds into representative point targets. Next, the prediction module generates prior state estimates for existing tracks, and the data association module matches the predicted tracks with current measurements. Finally, the state update module estimates the target states and outputs the tracking results.

The proposed pipeline includes point cloud clustering, data association, and track filtering. Based on the limitations of conventional methods and the practical challenges of extended-target tracking, such as an unknown number of targets and difficulty separating closely spaced targets, this paper improves the clustering and association modules and validates the resulting algorithm using measured radar data.

### 4.2. Traditional DBSCAN Clustering Algorithm

DBSCAN is a density-based clustering algorithm that does not require the number of clusters to be specified in advance. Instead, it clusters data based on the density of sample points and is capable of discovering clusters of arbitrary shapes. The algorithm defines two important concepts: the ε-neighborhood and core points. The ε-neighborhood refers to all points within a radius of ε centered on a given point; a core point is a point whose ε-neighborhood contains a number of points exceeding a preset threshold MinPts. The basic steps of the DBSCAN algorithm are as follows:

1. Calculate the ε-neighborhood of all points. For each point in the dataset, compute how many data points fall within its ε-neighborhood. The threshold for the number of points is typically defined by the parameter MinPts.
2. Mark core points. If the number of points in a point’s ε-neighborhood is greater than or equal to MinPts, the point is marked as a core point.
3. Find density-connected points. For each core point, find all points that are density-connected to it. If point *P* is within the ε-neighborhood of point *O* and *O* is a core point, then *P* is density-connected to *O*.
4. Mark noise points and border points: Points that are not marked as core points are labeled as noise points. Points that are density-connected to a core point but are not themselves core points are labeled as border points.
5. Assign an independent cluster label to each core point and its density-connected points. If a point is density-connected to multiple core points, it receives the cluster label of the first core point found.
6. Noise points form an independent cluster: All noise points constitute a separate cluster.

Although DBSCAN can identify clusters with arbitrary shapes and is robust to sparse noise, it also has several limitations. First, the algorithm is sensitive to the selection of ε and MinPts. Improper parameter settings can lead to missed clusters, false clusters, or over-clustering, and so the parameters must usually be tuned according to measured data.

Second, standard DBSCAN has difficulty separating closely spaced targets. When two targets are near each other, their point clouds may become density-connected. Because the algorithm relies only on spatial density, it may merge the two targets into one cluster.

Third, standard DBSCAN ignores radar-specific features such as Doppler velocity and signal-to-noise ratio (SNR). Points belonging to the same physical target usually exhibit similar velocity characteristics, whereas points from different targets or multipath reflections may differ in velocity and signal quality.

Finally, although DBSCAN can reject isolated noise points, dense noise or multipath points may still form false clusters. When a target is close to a wall or a large obstacle, multipath reflections can generate dense point clouds near the target. If these false clusters are not identified, they may degrade subsequent association and tracking performance.

### 4.3. Improved DBSCAN Clustering Algorithm

For indoor extended-target tracking, standard DBSCAN has three main defects: it uses only spatial position and ignores velocity information; it does not constrain the physical extent of a cluster, which can merge multiple nearby targets; and it lacks an explicit mechanism for suppressing false clusters caused by multipath effects.

**Figure 5:**
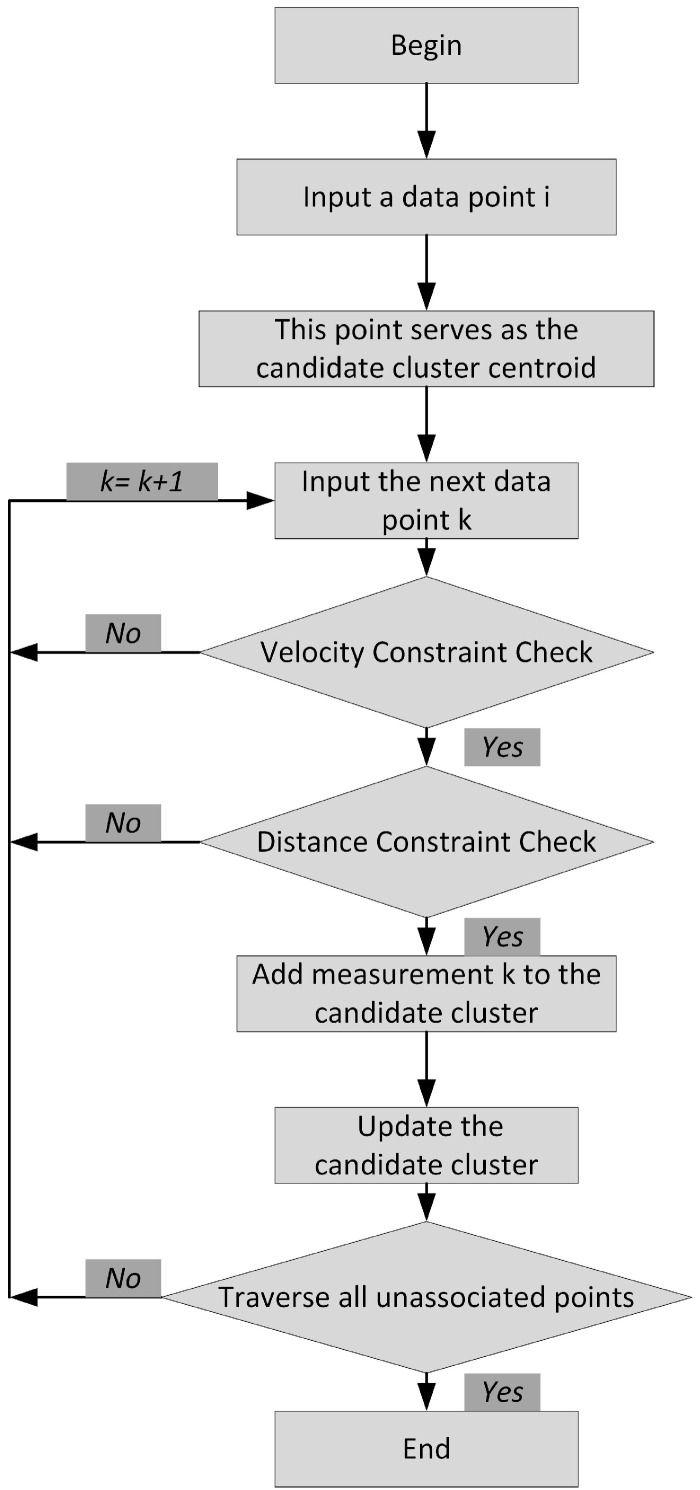
Flowchart of candidate cluster establishment.

To address these problems, this paper proposes an improved DBSCAN clustering algorithm based on a cluster-then-validate paradigm. Unlike methods that mainly adapt the DBSCAN radius or embed multiple features into a single distance metric, the proposed method separates density clustering from feature-based validation. In the first stage, a modified density-based procedure generates candidate clusters by combining spatial proximity with velocity consistency and spatial-extent constraints, as shown in Figure 5. In the second stage, each candidate cluster is validated by three interpretable checks: minimum point count, minimum velocity, and minimum SNR. Only clusters that pass all validation gates are confirmed as true targets. This structure incorporates indoor radar domain knowledge while adding little computational overhead, making it suitable for separating closely spaced human targets and suppressing false clusters in cluttered indoor environments.

#### 4.3.1. Implementation of the Improved DBSCAN Clustering Algorithm

In indoor environments, typical moving targets include adults, children, rotating fans, and robotic vacuum cleaners. Their radar point clouds occupy limited spatial extents in the *X* and *Y* directions. However, standard DBSCAN assigns points to clusters solely according to density connectivity and does not consider the physical size of the resulting cluster or the velocity similarity among its points. The improved clustering algorithm is therefore divided into two steps: (1) candidate cluster establishment and (2) candidate cluster detection.

(1) Establishment of candidate clusters

Candidate cluster establishment groups similar radar points into preliminary clusters. Compared with standard DBSCAN, the improved algorithm introduces three thresholds based on target point-cloud extent and motion consistency: (1) the maximum *x*-direction extent $X_{max}$; (2) the maximum *y*-direction extent $Y_{max}$; and (3) the maximum velocity spread $V_{max}$.

The flowchart of candidate cluster establishment is shown in Figure 5. The process begins with the preprocessed radar point cloud. Each unvisited point is treated as a potential seed point. For the current seed, the algorithm searches for neighboring points within the DBSCAN distance threshold ε, preserving the density-connectivity principle of standard DBSCAN.

Unlike standard DBSCAN, each neighbor must also pass two physical consistency checks. First, the radial velocity difference between the point and the current centroid velocity of the growing candidate cluster is calculated. If the difference exceeds $V_{max}$, the point is considered kinematically inconsistent and is skipped. Second, the coordinate differences between the point and the current cluster centroid are checked in the *X* and *Y* directions. If either difference exceeds the corresponding spatial extent limit, the point is rejected because it is unlikely to belong to the same finite-size indoor target.

Points that pass both checks are added to the candidate cluster. After each accepted point, the cluster centroid is updated using the current cluster members. This dynamic update makes subsequent checks rely on the latest estimated center of the candidate target. The process continues until all density-connected points around the seed are processed, and the resulting candidate cluster is passed to the validation stage.

(2) Detection of candidate clusters

**Figure 6:**
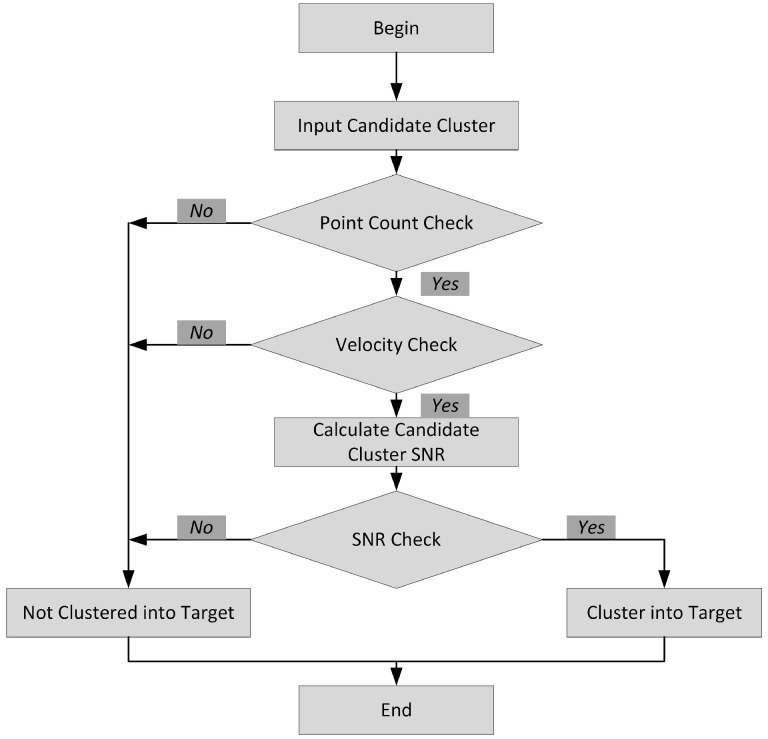
Flowchart of candidate cluster detection.

Candidate clusters may still be produced by noise or multipath points, and so a validation stage is required. Noise-induced clusters usually have fewer points, a lower SNR, and lower velocity than clusters generated by real moving targets. Three validation thresholds are therefore added: (1) the minimum point count $P_{min}$; (2) the minimum velocity $V_{min}$; and (3) the minimum signal-to-noise ratio ${SNR}_{min}$. The flowchart of candidate cluster detection is shown in Figure 6.

For each candidate cluster, the number of contained points is first counted. If it is below $P_{min}$, the cluster is rejected as too sparse. The centroid velocity is then checked. If its absolute value is below $V_{min}$, the cluster is regarded as nearly stationary or clutter-like and is rejected in the moving-target tracking pipeline. Finally, the average SNR of all points in the cluster is calculated. If it is below ${SNR}_{min}$, the cluster is rejected because it is likely caused by weak multipath reflections or noise rather than a real target. A candidate cluster that passes all three checks is confirmed as a valid target cluster, and its centroid is output as a representative point target for subsequent tracking. Otherwise, it is labeled as noise and discarded.

#### 4.3.2. Performance Validation of the Improved DBSCAN Clustering Algorithm

To validate the feasibility of the improved DBSCAN clustering algorithm, simulation experiments are conducted on a platform equipped with an AMD Ryzen 7 3700X 8-core processor (3.60 GHz) and 32 GB memory.

(1) The influence of different parameter values on the improved DBSCAN clustering algorithm

**Figure 7:**
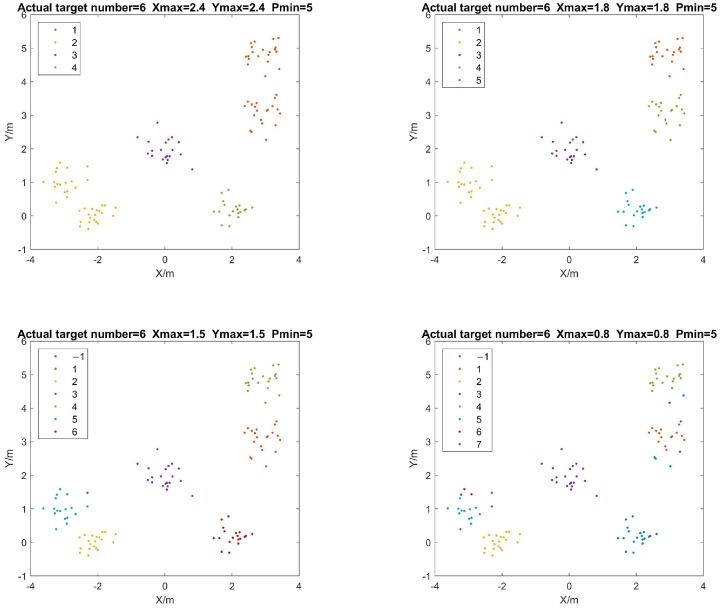
Clustering results for different values of $X_{max}$ and $Y_{max}$.

Six targets are randomly generated on a two-dimensional plane, and 20 data points are distributed around each target. The points follow Gaussian distributions whose means are the target positions. In addition, 40 interference points are randomly distributed over the plane. The parameters $X_{max}$, $Y_{max}$, and $P_{min}$ are determined through empirical tuning guided by physical prior knowledge. Initial values of $X_{max}$ and $Y_{max}$ are selected according to the spatial extent of typical indoor human targets, while $P_{min}$ is selected according to radar point-cloud density. First, $P_{min}$ is fixed at 5, and $X_{max}$ and $Y_{max}$ are varied to evaluate the influence of spatial constraints on clustering performance. The simulation results are shown in Figure 7.

**Figure 8:**
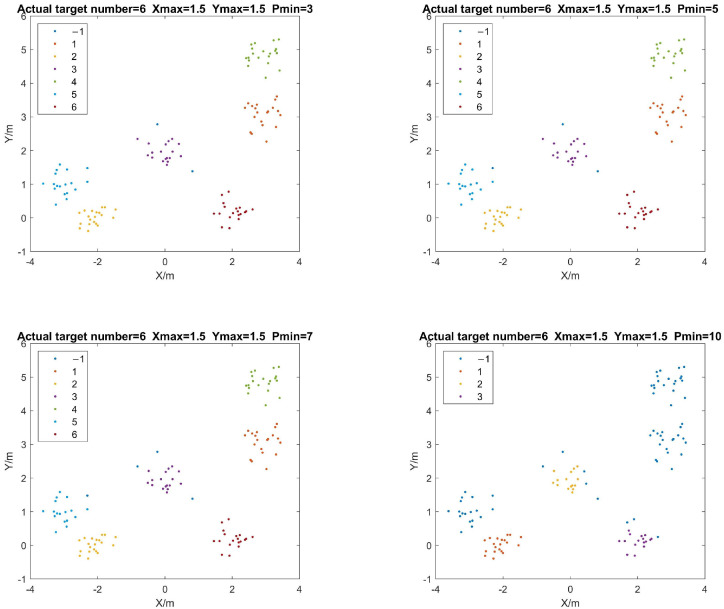
Clustering Results with Different Values of $P_{min}$.

As shown in Figure 7, the six targets are correctly clustered when $X_{max}$ and $Y_{max}$ are both set to 1.5 m. If the spatial thresholds are too small, a single target may be split into multiple clusters. If they are too large, nearby targets may be merged. Accordingly, $X_{max}$ and $Y_{max}$ are fixed at 1.5 m, and $P_{min}$ is varied to evaluate the influence of the minimum point-count threshold. The results are shown in Figure 8.

Figure 8 shows that the clustering results are similar when $P_{min}$ is set to 5 or 7. Because each simulated target contains sufficient points, $P_{min}=7$ is selected to strengthen noise suppression without sacrificing target detection. The final parameter set, $X_{max}=Y_{max}=1.5$ m and $P_{min}=7$, is fixed for the subsequent experiments. This selection process ensures that the parameters are physically meaningful and suitable for indoor multiple-extended-target tracking.

(2) Comparison between the traditional DBSCAN algorithm and the improved DBSCAN algorithm

**Figure 9:**
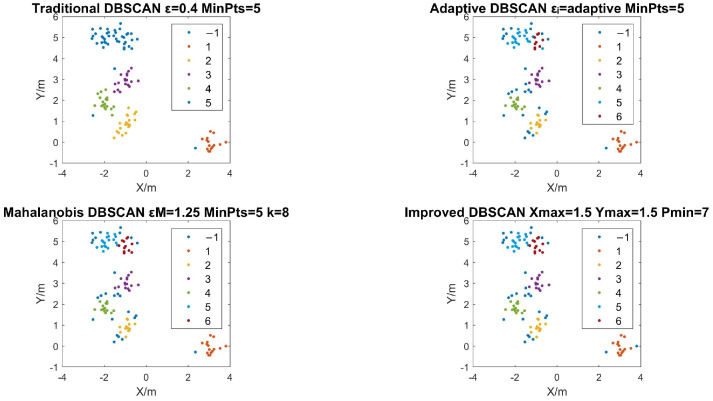
Clustering Results for Different Clustering Algorithms.

Using the parameter settings determined above, the same simulated point clouds are processed by traditional DBSCAN, adaptive DBSCAN [28], Mahalanobis distance-based DBSCAN [30], and the proposed improved DBSCAN. Each experiment is repeated 30 times. The clustering results are shown in Figure 9, and the statistical comparison is given in Table 2.

Figure 9 and Table 2 show clear differences among the four algorithms. Traditional DBSCAN (ϵ=0.4,MinPts=5) detects only 4.87 targets on average because it cannot reliably handle density variation or separate closely spaced targets. Adaptive DBSCAN improves robustness by estimating a local neighborhood radius from *k*-nearest-neighbor information, increasing the average number of detected targets to 5.93. Mahalanobis distance–based DBSCAN further improves shape adaptability by using covariance information to describe non-spherical clusters, achieving an average of 5.98 detected targets. The proposed improved DBSCAN ($X_{max}=Y_{max}=1.5$ m, $P_{min}=7$) achieves 5.97 detected targets on average and maintains stable separation through the cluster-then-validate strategy.

The computational costs also differ. For six targets, traditional DBSCAN takes 0.0965 s, adaptive DBSCAN takes 0.1174 s because of additional KNN preprocessing, and Mahalanobis distance–based DBSCAN takes 0.2034 s because covariance estimation and distance transformation are computationally expensive. In contrast, the proposed improved DBSCAN takes 0.0974 s, only slightly longer than the traditional baseline. It therefore provides a better balance between detection accuracy and computational efficiency for real-time indoor radar tracking.

To further evaluate computational efficiency and scalability, supplementary benchmarks are conducted for scenarios containing 2 to 10 targets. Table 3 summarizes the average number of points and the average processing time of each algorithm.

The benchmark results show that the four algorithms have different trade-offs. Traditional DBSCAN provides the lowest baseline processing time but is unreliable when target density changes or targets are close together. Adaptive DBSCAN and Mahalanobis distance–based DBSCAN improve clustering accuracy, but they increase computation because of adaptive radius estimation, covariance calculation, and distance transformation. Mahalanobis distance–based DBSCAN is especially suitable for elongated targets and anisotropic point clouds, such as vehicles in road scenes, but its computational cost is less favorable for embedded real-time indoor systems. The proposed improved DBSCAN exploits the known physical extent of indoor targets and validates candidate clusters after density grouping. As a result, it achieves high detection accuracy with almost no additional processing cost, making it more suitable for real-time indoor multi-person tracking.

### 4.4. Optimized NNDA Data Association Algorithm

When millimeter-wave radar detects multiple targets in a real indoor scene, it generates many point-cloud measurements. These measurements may originate from true targets, noise, or false alarms. Data association determines which measurements should be assigned to existing tracks so that each track is updated by measurements most likely belonging to the same physical target.

The association algorithm assigns measurements according to a distance metric between current points and predicted tracks, while marking measurements that cannot be assigned. Unassociated measurements in the current frame are collected as potential new-target evidence. In subsequent processing cycles, they are passed to the clustering and initiation modules together with new detections; if they form persistent clusters over time, new tracks can be initiated.

Because NNDA has low computational complexity, a simple structure, and good engineering implementability, it is selected as the basis of the association module and then optimized for extended radar targets.

**Figure 10:**
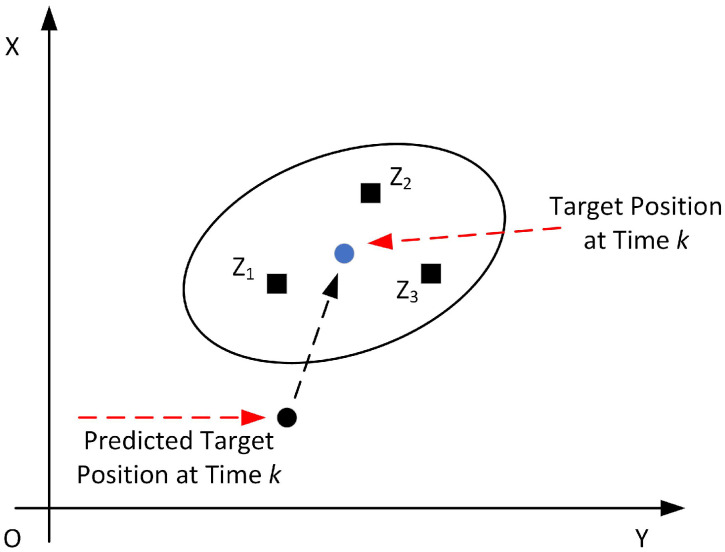
Gate size.

NNDA associates each track with the nearest valid measurement. A tracking gate is first centered on the predicted target position from the previous frame. The gate size is selected so that true target measurements fall inside it with high probability. Measurements inside the gate are treated as candidates. If multiple candidates exist, the measurement with the smallest statistical distance is selected for association, as illustrated in Figure 10.

A measurement point falling within the gate should satisfy the following formula:

(13) $${\left(Z\left(k+1\right)-\hat{Z}\left(k+1|k\right)\right)}^{⊤}S^{-1}\left(k+1\right)\left(Z\left(k+1\right)-\hat{Z}\left(k+1|k\right)\right)\leq G$$

where the measurement point Z(k+1) is considered a candidate association for a track if its normalized innovation squared relative to the predicted measurement $\hat{Z}\left(k+1|k\right)$ is less than or equal to the threshold *G*. The statistical distance between the measurement and the gate center is

(14) $$d^{2}\left(Z\right)={\left(Z\left(k+1\right)-\hat{Z}\left(k+1|k\right)\right)}^{⊤}S^{-1}\left(k+1\right)\left(Z\left(k+1\right)-\hat{Z}\left(k+1|k\right)\right)$$

Among all candidate measurements falling within the gate, the one with the smallest $d^{2}$ is selected for association in the standard NNDA rule.

The main weakness of standard NNDA is that the nearest candidate is not always the correct one in complex scenes, especially when targets are close to each other or their trajectories cross. This can cause false association, target loss, or track switching.

Standard NNDA is mainly designed for point targets, where each target produces a single measurement in each frame. For extended targets, however, each target may generate multiple radar points. A common solution is to cluster the point cloud first and then associate each cluster centroid with an existing track, but this increases processing stages and time cost.

**Figure 11:**
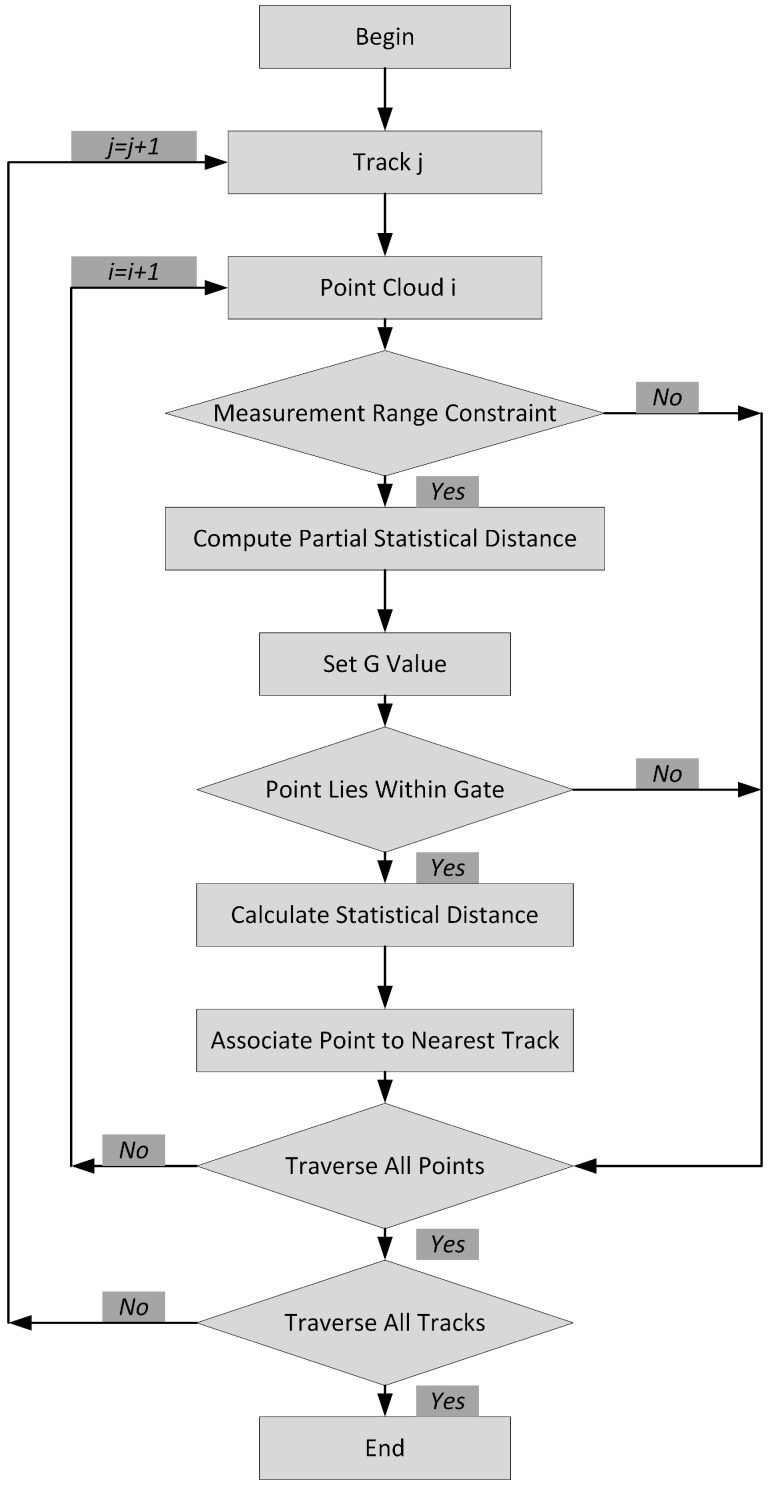
Schematic diagram of the NNDA algorithm.

Here, NNDA is integrated with clustering constraints. Existing tracks are used to associate raw point-cloud measurements, and spatial and kinematic restrictions are introduced during association. This realizes data association for extended target point clouds while reducing unnecessary processing. The algorithm flow is shown in Figure 11.

The process consists of the following key steps:

1. Input and initialization. The algorithm starts with the current radar measurement points and the list of existing tracks, whose predicted states are provided by the EKF.
2. Physical constraint pre-filtering. Each potential point-track pair is first checked using simple distance, angle, and velocity limits. Pairs that violate these constraints are discarded before statistical gating, reducing computational load.
3. Statistical gating. For pairs passing the pre-filter, the statistical distance is calculated. A measurement is considered a valid candidate for a track only when its statistical distance is smaller than the gate threshold *G*.
4. Nearest-neighbor selection. For each track, the valid candidate with the smallest statistical distance is selected as the best match in the current frame.
5. Global association and conflict resolution: All point–track pairs are traversed, and conflicts are resolved to maintain one-to-one associations where possible. Confirmed point–track pairs are used for EKF update, while unassociated points are reserved for possible initiation of new tracks.

### 4.5. Track Prediction and Updating Based on the EKF

The EKF based on the CA motion model is used for track prediction and update. For the two-dimensional tracking scenario, the target state vector is $X={\left[x,\dot{x},\ddot{x},y,\dot{y},\ddot{y}\right]}^{T}$, and the measurement vector is $z={\left[\rho ,\phi \right]}^{T}$, where ρ is the radial distance and ϕ is the azimuth angle. The nonlinear measurement function from the state vector to the measurement vector is

(15) $$h\left(X\right)=\left[\begin{array}{c} \sqrt{x^{2}+y^{2}}\\ \arctan2(x,y) \end{array}\right]$$

The Jacobian matrix is obtained by taking the first-order partial derivative of the measurement function

(16) $$H=\frac{\partial h}{\partial X}=\left[\begin{array}{cccccc} \frac{x}{\rho } & 0 & 0 & \frac{y}{\rho } & 0 & 0\\ \frac{y}{\rho^{2}} & 0 & 0 & -\frac{x}{\rho^{2}} & 0 & 0 \end{array}\right]$$

where $\rho =\sqrt{x^{2}+y^{2}}$. On the basis of the above Jacobian matrix, the optimal estimation of the target state can be completed through the EKF prediction and update steps described in Section 3.3.2.

## 5. Experimental Analysis and Results

### 5.1. Experimental Platform and Parameter Settings

**Figure 12:**
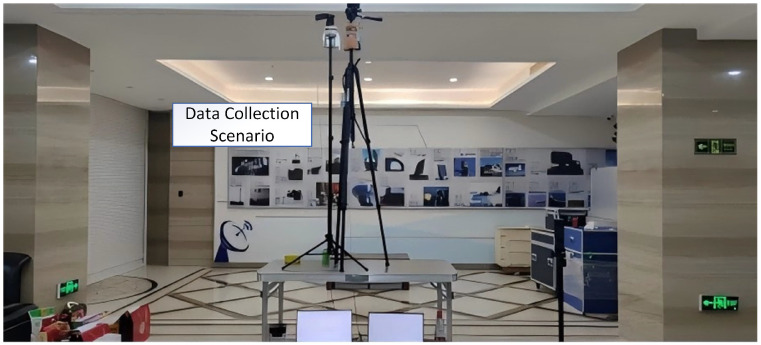
Data collection Scenario.

**Figure 13:**
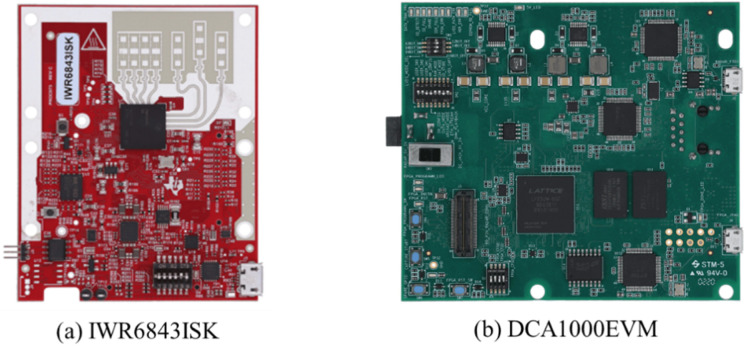
Data collection platform.

The experiments are conducted in a typical indoor home environment with an area of 6 m × 6 m. The data acquisition platform consists of a TI IWR6843ISK millimeter-wave radar evaluation board and a DCA1000EVM data acquisition board, as shown in Figure 12 and Figure 13. The core radar parameters are listed in Table 4, and the corresponding radar performance indicators are listed in Table 5. In the following experiments, the tracking targets are adult human subjects. The physical dimensions of a typical adult, such as a shoulder width of approximately 0.4–0.5 m and a height of approximately 1.6–1.8 m, are considered when setting the spatial clustering parameters. Ground-truth trajectories are obtained using a high-precision ultra-wideband (UWB P440 Module) positioning system. The UWB system provides real-time three-dimensional coordinates with centimeter-level accuracy and serves as the reference trajectory. Synchronization is achieved through a shared software trigger. A central control PC sends a simultaneous start command to both the radar data logging software and the UWB recording software. The two systems timestamp their data using internal clocks synchronized at the beginning of each experimental session through a network time protocol. In the following comparison, “Our System” denotes the TI IWR6843ISK hardware running the proposed tracking pipeline, whereas “TI System” denotes the same hardware running the built-in on-chip tracking firmware.

### 5.2. Single-Person Tracking Accuracy Experiment

Single-person data are collected under different motion scenarios. EKF-based tracking is then performed, and the tracking errors are analyzed to evaluate the effectiveness of the proposed algorithm.

#### 5.2.1. Linear Motion Scenario

**Figure 14:**
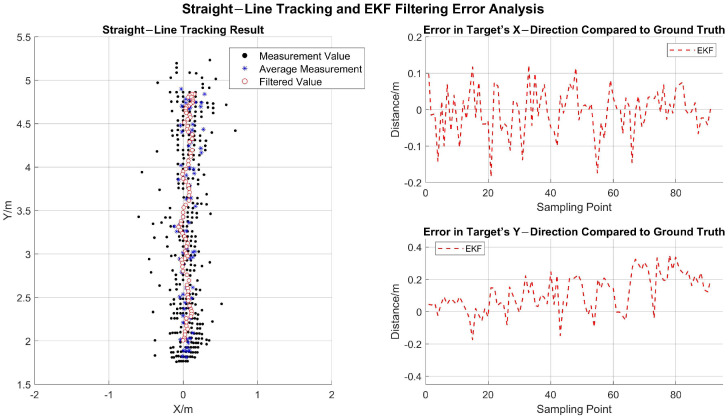
Linear motion tracking results and error analysis.

The linear motion tracking result is shown in Figure 14. The target moves slowly toward the radar along a straight line from a position 5 m away. The tracked trajectory is consistent with the actual motion, and the filtered trajectory is continuous and smooth. The tracking error is within 0.2 m in the X direction and within 0.4 m in the Y direction. Because the human body is an extended target, different body parts generate radar points at different ranges. When the target approaches or moves away from the radar, this range variation is mainly reflected in the Y direction, resulting in a slightly larger Y-direction error. Nevertheless, the overall error remains acceptable.

#### 5.2.2. Circular Motion Scenario

**Figure 15:**
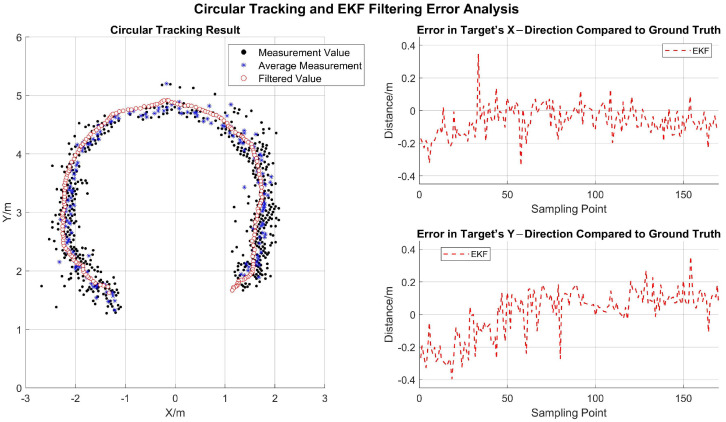
Circular motion tracking results and error analysis.

The circular motion tracking result is shown in Figure 15. The proposed algorithm maintains stable tracking when the target moves along a circular trajectory. In this case, the range differences among radar points from different body parts affect both the X and Y directions. The tracking errors in both directions remain within 0.4 m, indicating reliable tracking performance.

#### 5.2.3. Figure-Eight Motion Scenario

**Figure 16:**
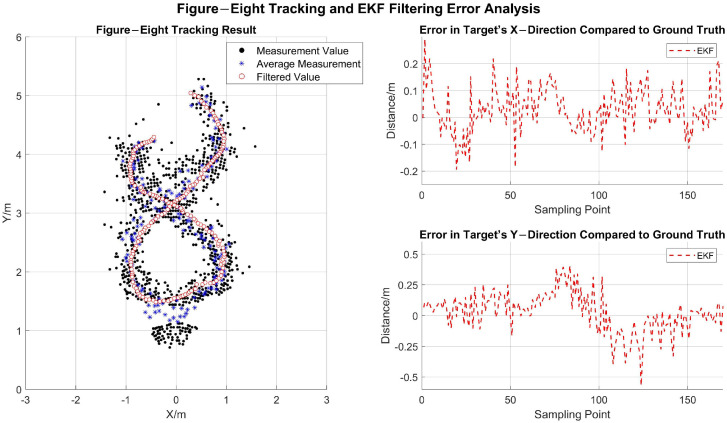
Figure-Eight motion tracking results and error analysis.

The figure-eight motion tracking result is shown in Figure 16. The algorithm also maintains stable tracking under this more complex trajectory. When the target is close to the radar, several interference points appear, but the filtered trajectory remains consistent with the actual motion. The tracking errors in both the X and Y directions are within 0.4 m, demonstrating robustness to moderate interference.

The above results show that the proposed tracking algorithm can handle common indoor motion patterns. The estimated trajectories closely follow the reference trajectories with small deviations, indicating that the algorithm is effective for moving-target tracking in home scenarios.

#### 5.2.4. Dual-Person Crossing Motion Scenario

**Figure 17:**
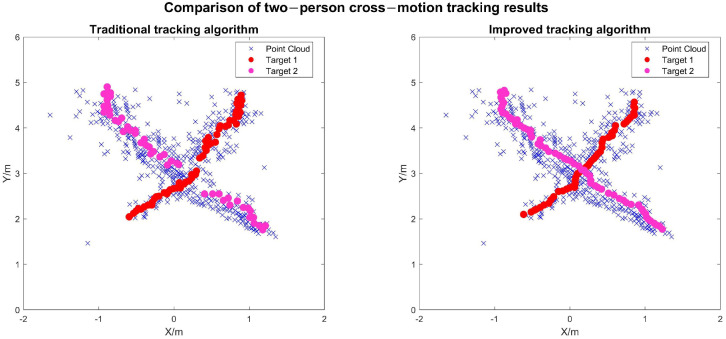
Dual-person crossing tracking results.

Dual-person crossing experiments are conducted, and 150 frames of data are collected. The measurements are processed using both the traditional clustering-and-tracking method and the proposed improved method. The tracking results are shown in Figure 17, and the statistical results are listed in Table 6.

Table 6 shows that the traditional tracking algorithm is less reliable in the dual-person crossing scenario. When the two targets approach each other, the traditional method loses one target in several frames. In contrast, the proposed method maintains continuous tracking of both targets, demonstrating better robustness in indoor crossing scenarios.

#### 5.2.5. Analysis of Real Scenario Execution Time

This subsection reports the execution time of the proposed pipeline in the scenarios described above, including single-person linear motion, circular motion, figure-eight motion, and dual-person crossing. Table 7 lists the average execution time of each major module and the total processing time per frame, benchmarked against the radar frame period.

The measured results show that the total per-frame processing time in all tested scenarios is 8.12–11.33 ms, which is much lower than the radar frame period of 80 ms. The proposed pipeline therefore has sufficient timing margin for real-time operation.

### 5.3. Multi-Person Tracking Experiment

The multi-person tracking experiment includes two evaluations: person-counting accuracy and Circular Error Probable (CEP). Person-counting accuracy measures whether the system can stably track the correct number of people in the scene, while CEP quantifies the positioning error between the estimated trajectory and the reference trajectory.

#### 5.3.1. Person-Counting Accuracy Experiment

**Figure 18:**
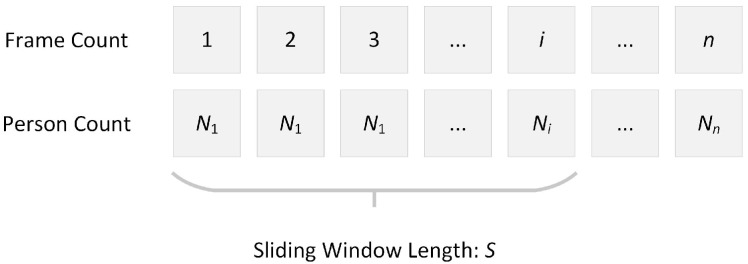
Sliding window.

For a multi-person tracking system, the accuracy of real-time person counting is an important performance indicator. It is calculated as the percentage of frames in which the estimated number of targets equals the actual number of people present. To reduce the influence of transient errors, a sliding-window method is used, as shown in Figure 18.

Let $N_{i}$ denote the number of people estimated in frame *i*, and let *S* denote the sliding-window length. When i<S, the most frequent estimated count among the first *i* frames is used as the count for the current frame. When i≥S, the most frequent estimated count within the window from frame i−S+1 to frame *i* is used. The counting accuracy is then calculated as the ratio of frames whose smoothed count equals the true number of people to the total number of processed frames.

**Figure 19:**
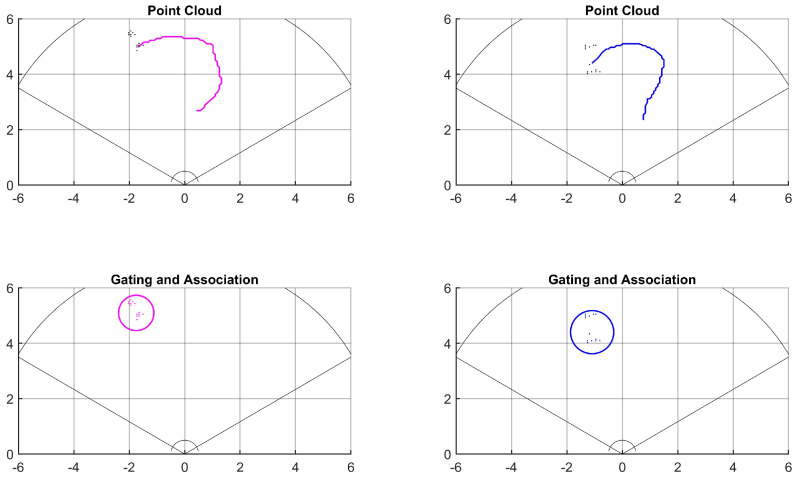
Single-person motion tracking results.

In the single-person movement scenario experiment, the person walks freely at any position in the scene; the results are shown in Figure 19.

Figure 19 shows that both systems can detect and continuously track a single person, and the estimated trajectories are similar. The distribution of estimated person counts is shown in Table 8.

Table 8 indicates that the proposed system detects and tracks a single person with 100% counting accuracy. The TI system occasionally estimates two persons, but its overall single-person performance is still close to that of the proposed system.

**Figure 20:**
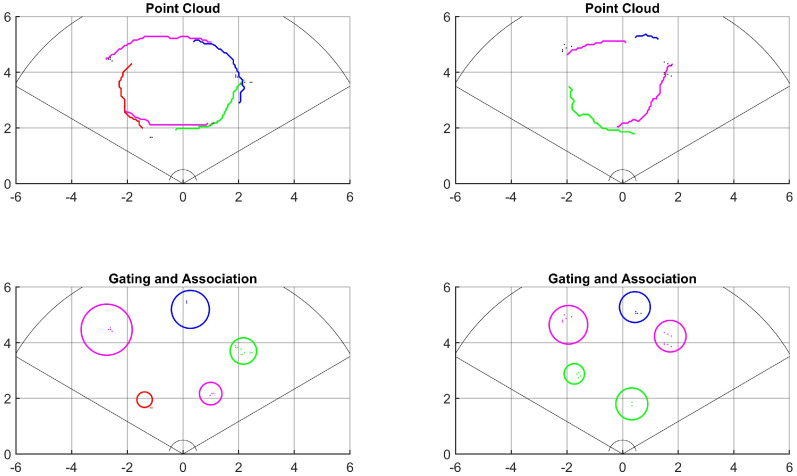
Multi-person motion tracking Results.

In the multi-person scenario, the participants maintain a certain distance from each other to avoid complete occlusion, because severe occlusion reduces the quality and quantity of radar points and affects tracking performance. Figure 20 and Table 9 present the person-counting performance when the true number of people is five. The column headers 3, 4, 5, and 6 denote the estimated counts, and the table values indicate the percentage of frames assigned to each count. The results show that the proposed system correctly estimates five people in most frames, and most errors are one-person undercounts or overcounts.

The results show that mutual interference among targets is significant in multi-person motion. Point clouds from different targets may overlap or merge, reducing point-cloud quality and causing occasional undercounting. Nevertheless, the proposed system loses targets only in a few cases and achieves higher counting accuracy than the TI system.

#### 5.3.2. CEP Experiment

The CEP is a metric that measures the accuracy of a positioning system. It is defined as the radius of the circle, centered on the ground truth position, within which 50% of the estimated positions are expected to fall. A smaller CEP indicates higher tracking accuracy.

**Figure 21:**
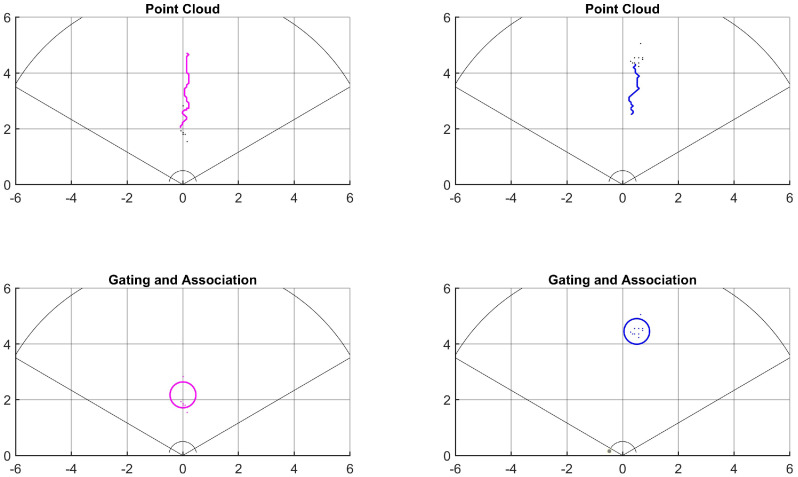
Single-Person CEP Experiment Results.

In the single-person CEP experiment, the person walks along the straight line x=0 m. The results are shown in Figure 21 and Table 10.

The results show that the estimated trajectory fluctuates around the true straight-line path. This is mainly because the human body is an extended target; radar points may be generated from different body parts. Body shape and walking posture also influence the point-cloud distribution; for example, large arm swings can increase trajectory fluctuation. The proposed system suppresses these effects more effectively than the TI system does, producing a trajectory that is closer to the reference path and achieving higher tracking accuracy.

## 6. Conclusions

To meet the perception requirements of indoor smart home scenarios, this paper proposes a multiple-extended-target tracking algorithm based on 60 GHz millimeter-wave radar. The method avoids the illumination sensitivity, occlusion sensitivity, and privacy risks of vision-based perception while improving the robustness of radar tracking in cluttered indoor environments. This study’s main contributions to the literature are summarized as follows:

(1) An improved DBSCAN clustering algorithm is proposed. By introducing spatial-extent constraints, velocity-consistency constraints, and candidate-cluster validation, the algorithm reduces cluster merging for closely spaced targets and suppresses false targets caused by multiple paths while adding little computational complexity.

(2) An optimized NNDA data association algorithm is developed. By integrating extended-target clustering constraints into the association process, the method improves real-time performance and association accuracy.

The proposed system is validated through real-world experiments. In typical single-person motion scenarios, the tracking error is controlled within 0.4 m. In a two-person crossing scenario, continuous tracking of both targets is maintained. In a five-person scenario, the person-counting accuracy reaches 93.3%. Overall, the proposed system outperforms the commercial TI radar tracking system under the tested indoor conditions.

These results indicate that the proposed method can provide a reliable and privacy-preserving tracking solution for smart homes, elderly care, indoor security, and related intelligent sensing applications.

## Figures and Tables

**Table 1:** Representation in different coordinate systems.

Global Coordinate System	$\left[X_{w},Y_{w},Z_{w}\right]$	Rectangular coordinate system with the origin at a reference point on the ground.
Local Coordinate System	$\left[X_{t},Y_{t},Z_{t}\right]$	Rectangular coordinate system with the origin at the radar antenna position.
Local Polar Coordinate System	[ρ,ϕ,θ]	Polar coordinate system with the origin at the radar antenna position.
Radar Position Coordinates	$\left[0,0,H_{r}\right]$	$H_{r}$ is the height of the radar above the ground.

**Table 2:** Statistical comparison for different clustering algorithms.

Algorithm	Average Algorithm Time (s)	Average Number of Clustered Targets
Traditional DBSCAN	0.0965	4.87
Adaptive DBSCAN	0.1174	5.93
Mahalanobis DBSCAN	0.2034	5.98
Improved DBSCAN	0.0974	5.97

**Table 3:** Comprehensive performance of different clustering algorithms.

Targets	Average Points	Average Algorithm Time (s)			
		Traditional	Adaptive	Mahalanobis	Improved
2	60	0.0405	0.0514	0.1045	0.0394
3	81	0.0560	0.0699	0.1332	0.0570
4	108	0.0744	0.0923	0.1672	0.0776
5	135	0.0933	0.1133	0.1993	0.0968
6	162	0.0965	0.1174	0.2034	0.0974
8	216	0.1579	0.1899	0.3171	0.1602
10	270	0.1838	0.2191	0.3604	0.1849

**Table 4:** Radar core parameter settings.

Parameter	Value
Start Frequency (GHz)	60.6
Chirp Slope (MHz/µs)	53.011
Bandwidth (MHz)	3286.68
ADC Sampling Points	128
Frame Period (ms)	80
Sampling Rate (Ksps)	2500
Chirp Repetition Period (µs)	82
Number of Chirps	128
Number of Transmit Antennas	3
Number of Receive Antennas	4

**Table 5:** Radar resolution and maximum detection range.

Parameter	Value
Range Resolution (m)	0.0522
Maximum Unambiguous Range (m)	7.07
Velocity Resolution (m/s)	0.0681
Maximum Unambiguous Velocity (m/s)	15.09
Azimuth Resolution (°)	15
Azimuth Angle Range (°)	(−60, 60)

**Table 6:** Person-counting accuracy statistics (1–2 persons).

	Frame Count	Target 1	Target 1
Tracking Algorithm			
Traditional Tracking Algorithm		150	135
Improved Tracking Algorithm		150	150

**Table 7:** Per-frame execution time breakdown of the proposed algorithm pipeline.

Scenario	Points	Signal Proc. (ms)	Preproc. (ms)	Improved DBSCAN (ms)	NNDA + EKF + Trk (ms)	Total (ms)
Linear Motion	25	7.65	0.18	0.051	0.24	8.12
Circular Motion	28	8.23	0.20	0.058	0.24	8.73
Figure-Eight Motion	30	8.75	0.21	0.064	0.24	9.26
Dual-Person Crossing	55	10.47	0.39	0.138	0.33	11.33

**Table 8:** Distribution of estimated person counts (true count = 1).

	Number of People	1	2
System			
Our System		100.0%	0.0%
TI System		98.1%	1.9%

**Table 9:** Distribution of estimated person counts (true count = 5).

	Number of People	3	4	5	6
System					
Our System		0.0%	5.3%	93.3%	1.4%
TI System		4.4%	8.7%	86.1%	0.8%

**Table 10:** Single-person CEP error statistics.

Our System		TI System	
CEP 0.8 m	100%	CEP 0.8 m	100%
CEP 0.6 m	100%	CEP 0.6 m	100%
CEP 0.2 m	95%	CEP 0.29 m	95%
CEP 0.09 m	68%	CEP 0.16 m	68%

## Data Availability

The original contributions presented in this study are included in the article. Further inquiries can be directed to the corresponding author.

## Abbreviations

The following abbreviations are used in this manuscript:

CA	Constant Acceleration
CEP	Circular Error Probable
CV	Constant Velocity
DBSCAN	Density-Based Spatial Clustering of Applications with Noise
EKF	Extended Kalman Filter
FMCW	Frequency-Modulated Continuous Wave
JPDA	Joint Probabilistic Data Association
KF	Kalman Filter
MHT	Multi-Hypothesis Tracking
NNDA	Nearest Neighbor Data Association
PDA	Probabilistic Data Association
TI	Texas Instruments
SNR	Signal-to-Noise Ratio
